# Targeted Liposomal Drug Delivery: Overview of the Current Applications and Challenges

**DOI:** 10.3390/life14060672

**Published:** 2024-05-24

**Authors:** Matthew S. Gatto, McNeely P. Johnson, Wided Najahi-Missaoui

**Affiliations:** Department of Pharmaceutical and Biomedical Sciences, College of Pharmacy, University of Georgia, Athens, GA 30602, USA; matthew.gatto@uga.edu (M.S.G.); mcneely.johnson@uga.edu (M.P.J.)

**Keywords:** liposomes, drug delivery, targeted therapy, nanoparticles, active targeting, passive targeting, EPR, PEGylation

## Abstract

In drug development, it is not uncommon that an active substance exhibits efficacy in vitro but lacks the ability to specifically reach its target in vivo. As a result, targeted drug delivery has become a primary focus in the pharmaceutical sciences. Since the approval of Doxil^®^ in 1995, liposomes have emerged as a leading nanoparticle in targeted drug delivery. Their low immunogenicity, high versatility, and well-documented efficacy have led to their clinical use against a wide variety of diseases. That being said, every disease is accompanied by a unique set of physiological conditions, and each liposomal product must be formulated with this consideration. There are a multitude of different targeting techniques for liposomes that can be employed depending on the application. Passive techniques such as PEGylation or the enhanced permeation and retention effect can improve general pharmacokinetics, while active techniques such as conjugating targeting molecules to the liposome surface may bring even further specificity. This review aims to summarize the current strategies for targeted liposomes in the treatment of diseases.

## 1. Introduction

Bringing a new drug to the market can cost between 1 and 2 billion US Dollars over the span of 10–15 years [1], and lead compounds face long odds of being approved by the Food and Drug Administration (FDA). Over 90% of investigational drugs fail to receive FDA approval after they have already reached the clinical phases of development [2]. In some cases, these failures can be attributed to a poor commercial and operational outlook for the drug sponsor. However, a study conducted from 2013–2015 shows that 76% of these failures are the result of poor safety and efficacy [3]. Furthermore, even when a drug is approved, there are often off-target side-effects that are a cause for concern. In the case of cancer, common treatment options such as chemotherapy, immunotherapy, and radiation therapy are known to lead to harmful side effects including neuropathy, suppression of bone marrow, fatigue, and autoimmune disease [4]. For this reason, there has been an increased focus on utilizing drug delivery mechanisms to both reduce off-target toxicity and enhance overall efficacy.

These drug delivery mechanisms include nanoparticles such as liposomes. Liposomes are spherical vesicles consisting of an aqueous core bound by a phospholipid bilayer membrane. They can come in a wide range of sizes, but a diameter of 50–200 nm is typically desired for their drug delivery applications [5]. As of 2023, there have been 15 liposomal drug products approved by the FDA, and their indications include a wide range of applications including cancer treatments, vaccines, infections, and pain management (Table 1) [6].

There are several different methods by which liposomes can be synthesized. Perhaps the most common method is thin-film hydration. In this procedure, the lipid and drug components of the liposomes are mixed in an organic solvent that is removed over time through rotary evaporation. Once a dry thin film has formed on the flask, the components are rehydrated in an aqueous medium at a temperature above the phase transition temperature of the lipids. At this point, liposomes have formed, but further processing techniques such as freeze-thaw cycles, sonication, or extrusion can be used to further homogenize the size and lamellarity of the liposomes [8]. Another common method of liposome synthesis is reverse-phase evaporation (REV). Through this method, large aqueous volumes can be encapsulated, making it a compatible process for packaging larger therapeutic molecules [9]. During REV, the lipid components are mixed in an organic solvent, and then the aqueous phase containing the drug is introduced, forming a water-in-oil emulsion [10]. The organic phase is removed through rotary evaporation, and the remaining aqueous solution contains the therapeutic liposomes. Once again, further processing can be utilized for homogenizing the size. While thin-film hydration and REV are the two primary methods of liposome preparation, there are several unconventional approaches that are used as well. Microfluidics involves the laminar flow of dissolved lipids into a flowing aqueous phase, resulting in the self-assembly of the liposome. The flow rates of each phase can be tuned to achieve a desired size distribution [11]. The lack of a need for further homogenization is ideal, but unfortunately, the process is not scalable. Another unconventional preparation method is the supercritical anti-solvent (SAS) method. Lipids dissolved in a supercritical fluid, usually carbon dioxide, are hydrated with a drug-containing aqueous phase. When the pressure is reduced, liposomes will form with exceptionally high encapsulation efficiencies [10]. Unfortunately, the high costs of this technique hold it back from becoming more popular. Additional methods of preparation, such as the heating method [12] and freeze-drying method [13], are also used, but none are as common as thin-film hydration or REV.

In addition to the method of preparation, several formulation factors can also affect the properties of liposomes. For example, including cholesterol in the lipid membrane at ratios between 30 and 50% can provide additional stability to the nanoparticle through increased membrane ordering [14,15]. Furthermore, including cholesterol in the membrane has also been shown to increase cellular uptake [16]. Aside from cholesterol, the hydration medium in which liposomes are stored is also important. Typically, liposomes are kept in aqueous solutions such as water, phosphate-buffered saline, or 5–10% sugar solutions. The hydration medium will usually depend on the composition and application of the liposomal formulation. For example, liposomes composed of highly charged phospholipids may form a viscous gel if hydrated in solutions with low ionic strength [17]. Therefore, a higher concentration of salts should be included in the hydration medium. A third formulation factor to consider is the inclusion of surfactants. It has been observed that increased concentrations of surfactants during formulation are associated with a smaller liposome size [18]. This may be due to decreased vesicle aggregation, which is critical for effective storage of the nanoparticles.

**Figure 1:**
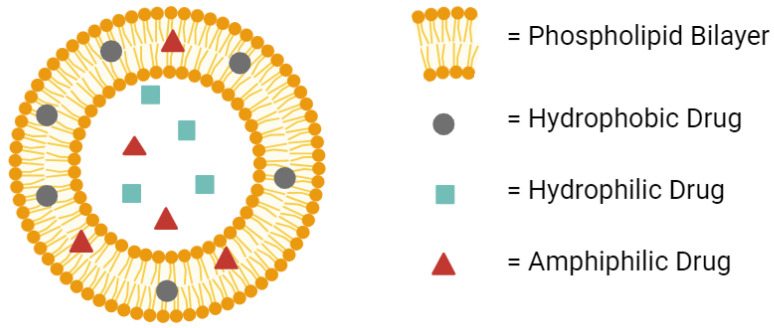
A conventional liposome can hold hydrophobic drugs in its lipid bilayer, hydrophilic drugs in its aqueous core, and amphiphilic drugs in either the bilayer or the core. Created with BioRender.com on 24 March 2024.

Liposomes provide many advantages for drug delivery. Their amphiphilic nature provides compatibility with both hydrophilic and hydrophobic drugs (Figure 1) [19]. Their low toxicity allows higher doses of the drug to be administered without compromising safety [20,21]. In fact, liposomes are considered among the least toxic nanoparticles available in the clinic [22]. Their improved half-life over the free drug provides the potential for extended-release dosage forms [23,24]. All of these advantages make liposomes worth the investment during drug development to avoid in vivo failures later on in clinical studies.

Perhaps the most valuable advantage of liposomes is the ability to customize their composition for targeted drug delivery. Many drugs, particularly in oncology and infections, are designed to be cytotoxic, and using these cytotoxic drugs comes with the obvious risk of damaging healthy cells in the body. Liposomes can be modified to be specific to the diseased cell or tissue and decrease off-target toxicity [25].

There are countless different diseased states that each requires a different approach to medical treatment. As a result, there are numerous strategies for utilizing liposomes as a drug delivery vehicle. This review aims to highlight the potential of the most widely investigated mechanisms for targeted liposomal therapy.

## 2. Passive Targeted Liposomes

While it is true that liposomes can be modified in many ways to achieve specific delivery, it is important to consider that even traditional liposomes can take advantage of physiological conditions in the body to preferentially deliver a drug to diseased tissue. This phenomenon is known as passive targeting, and there are a couple of different ways by which it occurs.

### 2.1. Enhanced Permeation and Retention Effect

**Figure 2:**
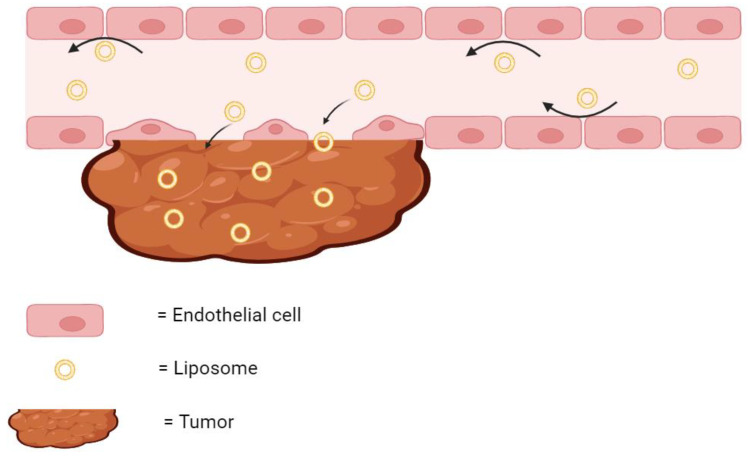
The EPR effect. Liposomes are too large to diffuse through healthy endothelial cells but readily diffuse through the leaky vasculature of tumor and accumulate. The arrows indicate the direction of the flow of liposomes. Created with BioRender.com on 24 March 2024.

As previously stated, many liposomal therapies have an indication for cancer. Tumors can be difficult to treat using traditional chemotherapy, as the microenvironment surrounding the cancerous tissue can possess disorganized and leaky vasculature, ineffective lymphatic vessels, and acute hypoxia [26,27,28]. All of these factors have been shown to reduce the delivery of chemotherapies to cancer cells [29,30]. However, encapsulating these drug molecules in liposomes creates a much bigger particle size. These bigger particles are less likely to extravasate through healthy blood vessels, and more likely to diffuse through the leaky vasculature of the tumor tissue (Figure 2). Therefore, while a free drug may be inadvertently delivered to healthy tissue in the body, liposomal drugs are much more likely to accumulate at the tumor site. Furthermore, the ineffective lymphatic drainage system results in the retention of these liposomes at the tumor site where they can continuously release their encapsulated drug [31]. This phenomenon of liposomes accumulating and remaining in the diseased tumor tissue is known as the Enhanced Permeation and Retention (EPR) Effect.

The EPR effect is a passive mechanism, and it can increase the efficacy of the encapsulated drug. It has been considered so critical to liposomal therapies that there is an interest in enhancing the EPR effect itself to improve drug performance. Liposomal cancer therapies can be given in tandem with liposomal nitric oxide donors. The addition of nitric oxide, a known vasodilator, results in an increased blood flow through the tumor vasculature. This increased blood flow allows more of the liposomal cancer therapy to penetrate the tumor tissue and release the drug substance. In one study, the accumulation of liposomes at the tumor site was doubled using this strategy [32]. In another example of EPR effect enhancement, liposomal daunorubicin was given after a form of cancer treatment known as photoimmunotherapy (PIT). PIT induces the death of perivascular tumor cells [33] which increases vascular leakage in the tissue. As a result of the increased leakage, more liposomal daunorubicin was able to find its way into the tumor. In fact, the researchers found there was an over 10-fold increase in the concentration of liposomal daunorubicin in the tumor when PIT was used in tandem with the liposomal treatment [34].

In modern drug development, the EPR effect alone is not always relied upon for efficacious drug delivery. However, as seen by the examples above, increasing the influence of the EPR effect can have a major impact on the specific delivery of liposomal drugs to cancerous tissue. This specificity remains a major justification for liposomal therapies over the free drug form.

### 2.2. Liposomes Conjugated with Polyethylene Glycol

**Figure 3:**
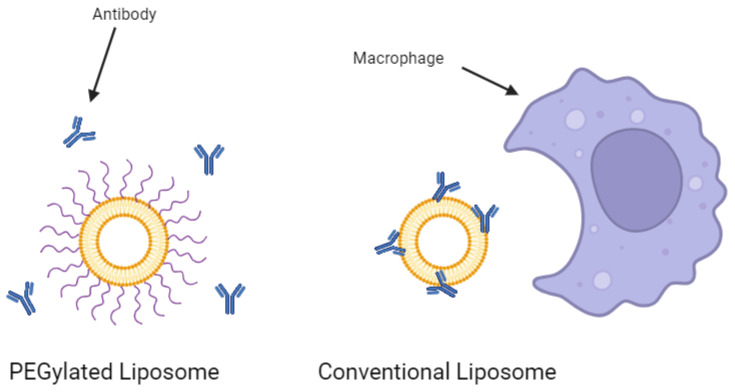
PEGylation of a liposome prevents antibody opsonization. Created with BioRender.com on 24 March 2024.

Conventional liposomes dramatically increase the half-life of a drug substance in the body [23]. However, they are still subject to removal from the bloodstream by the mononuclear phagocyte system (MPS) [35]. Macrophages work to remove the foreign liposomes over time, especially in key immune system organs such as the liver and spleen. The rate of clearance from the circulatory system can be dependent on various formulation factors such as the particle size, surface charge, and cholesterol content of the liposomes [36,37]. However, one of the most heavily researched strategies for improving circulatory stability is the conjugation of polyethylene glycol (PEG) to the surface of the liposomes. These so-called ‘stealth liposomes’ evade MPS uptake and can result in half-lives more than 3-fold that of conventional liposomes [38]. The PEG shield prevents antibodies and other proteins from opsonizing the liposomes (Figure 3), preventing immune action against the nanoparticles. A study investigating the effect of adding PEG to cisplatin-loaded liposomes to treat bladder cancer in mouse models showed that PEGylation resulted in a 4.8-fold increase in drug efficacy and a 3.3-fold decrease in drug toxicity over the non-PEGylated liposomes [39]. Furthermore, PEGylation was suggested to be crucial to the development of oral liposomal drug products. Singh et al. demonstrated that after 2 h in simulated gastric fluids, PEGylated liposomal formulations of exemestane leaked less than one-third the amount of drugs that non-PEGylated liposomes leaked [40]. Indeed, PEG has been recognized as highly effective in the delivery of liposomal drugs.

Attaching PEG to liposomes adds protection from the MPS, but the degree of protection is determined by several factors. There are numerous commercially available phospholipids that are conjugated with polyethylene glycol. The specific phospholipid used can impact vesicle stability, and therefore, the half-life of the liposome. The most commonly preferred lipid to conjugate with PEG is distearoyl-phosphatidylethanolamine (DSPE). DSPE’s longer acyl chains provide increased stability and integrity to the liposomes [41]. In addition to phospholipid choice, the length of the PEG polymer can have a significant impact on the liposomal stealth effect. The polymer chain can greatly vary in molecular weight, ranging from a few hundred Dalton up to several thousand Dalton. It has been demonstrated that longer PEG chains typically result in more favorable pharmacokinetics for liposomes [42]. This is because the hydrophilicity of PEG creates a protective aqueous layer around the liposome, and longer polymer chains will increase the thickness of this layer and provide a larger buffer from phagocytotic cells [43]. Another consideration that affects PEGylation efficacy is the method of insertion of PEG itself. PEG can be anchored to the liposomes either during formulation (pre-insertion PEGylation) or after formulation (post-insertion PEGylation). In the pre-insertion method, PEG strands protrude both inside and outside the bilayer membrane, whereas in the post-insertion method, the polymer is only present on the outside of the membrane. Several drug products have been approved using the pre-insertion method, carrying drug molecules such as doxorubicin, vincristine, and daunorubicin [44]. However, it is reported that using the post-insertion method may result in an increased PEG content, half-life, and encapsulation efficiency of liposomes as compared to the pre-insertion method [45,46]. Nonetheless, there is more research needed on this matter to reasonably evaluate the potential of one method over the other.

While stealth liposomes are used both commercially and in research, there is some concern over their repeated use. It has been demonstrated that if a second dose of PEGylated liposome is injected into a patient, it may have a shorter circulatory half-life than the first dose. This effect is known as the accelerated blood clearance (ABC) phenomenon, and it could make the PEGylation of nanoparticles counterproductive. The first exposure of PEG may lead to the production of anti-PEG antibodies by B cells. When the second dose of PEGylated liposomes is administered, these circulating antibodies bind to PEG on the liposome surface and activate the complement system, leading to phagocytosis by macrophages [47,48]. As a result, many of the liposomes do not reach their intended target. This reduces the efficacy of the treatment and creates an unfavorable biodistribution that could lead to toxic effects in the liver or other organs [49,50,51].

The effects of the ABC phenomenon are obviously significant, but it is worth mentioning that the ABC phenomenon is influenced by several characteristics of the liposome formulation. Unsurprisingly, the time interval between doses can have a major impact on the ABC phenomenon; the ABC effect generally decreases with increasing time between doses [52,53]. Furthermore, when smaller (<60 nm) liposomes are used in the first dose, the ABC phenomenon is enhanced upon the second dose, possibly due to the smaller liposomes’ preferential accumulation in the spleen where they interact with B cells [54]. Interestingly, Doxil^®^, one of two FDA-approved liposomal products using PEGylation, has not shown clinical signs of the ABC phenomenon [55]. Doxil^®^ is a liposomal formulation of doxorubicin, a cytotoxic drug used to treat several cancers. It is theorized that the high dose of doxorubicin carried in Doxil^®^ is highly cytotoxic to B cells in the spleen, leading to a much lower level of anti-PEG IgM antibodies produced than when empty liposomes are administered [56]. Only recently, at lower doses of encapsulated doxorubicin, were researchers able to observe the ABC phenomenon for Doxil^®^-like liposomes [57]. In other words, the off-target toxicity of high-dose Doxil^®^ in the spleen works to the drug’s advantage by preventing adaptive immunity against future Doxil^®^ doses. This is a troubling, yet interesting relationship, if future studies can confirm this mechanism.

All factors considered, PEGylation is an effective strategy for the passive targeting of liposomes. On the surface, its proven ability to increase the circulation time and efficacy of a drug-loaded liposome makes PEG seem like an easy formulation choice for liposomes. However, the potential induction of the ABC phenomenon requires that formulators consider the individual needs of their drug product before including PEG-conjugated phospholipids in their liposomes.

## 3. Active Targeted Liposomes

In addition to the previously mentioned passive targeting strategies, it is also possible to actively target specific sites in the body with liposomes. This can be accomplished by conjugating ligands, receptors, or small molecules to the surface of the liposomes to induce the preferential binding of the liposomes. Some of the well-known strategies for doing this are described below.

### 3.1. Antibodies

Antibodies are glycoproteins naturally produced by the immune system that recognize and bind to specific antigens [58]. They are composed of two heavy chains and two light chains, which together give antibodies their signature Y-shape. The stem of the Y, known as the fragment crystallizable (Fc) portion, is responsible for interactions between the antibodies and the immune cells that they anchor to. The two tips of the Y, known as the fragment-antigen-binding (Fab) portion, are responsible for the recognition of and attachment to the antigen [59]. When antibodies are activated by an antigen, there are several different mechanisms by which they respond, such as opsonization or complement system activation [60]. Their ability to both neutralize foreign threats and prompt further immune system activation makes them a powerful weapon in our bodies’ defense against diseases [61]. Biotechnology companies have acknowledged their promise, and as a result, eight new monoclonal antibodies were approved by the FDA in 2023 for commercial use [62].

**Figure 4:**
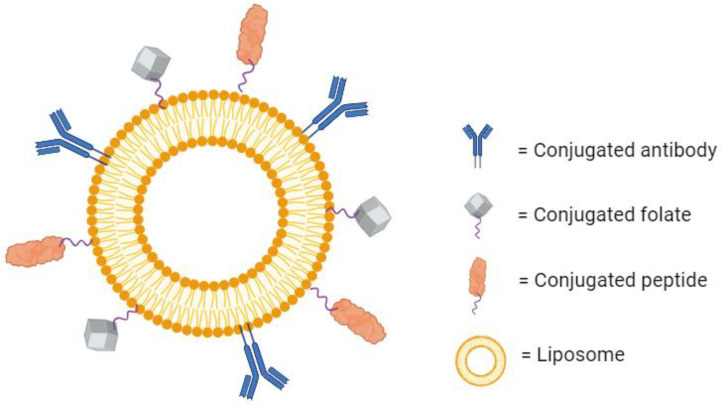
A liposome with several conjugated molecules for active targeting. Created with BioRender.com on 24 March 2024.

Beyond antibodies’ traditional therapeutic use, their specificity to one binding region can be utilized for targeted drug delivery. It is possible to conjugate antibodies to the surface of liposomes to create a highly specific drug-loaded nanoparticle (Figure 4). These are known as immunoliposomes (ILs), and they are an interesting point of research in targeted drug therapy. While the scientific rationale behind their development is sound, the efforts have not yielded definitive results. Some papers report a large increase in the specific accumulation of the liposomes over untargeted liposomes [63,64,65]. Additionally, some report no significant increase in local accumulation, but they do observe increased therapeutic effects as a result of increased cellular internalization [66]. On the other hand, several studies have also shown no substantial improvement in therapeutic effect over untargeted liposomes at all [67,68]. These mixed findings could be a result of increased clearance due to larger size, increased drug leakage, or poor stability in circulation [69]. For now, there are no immunoliposomes available commercially [70]. However, there are several in the preclinical or early clinical phases of development [71].

Recently, the strategy of conjugating whole antibodies to liposomes has been challenged. It seems that using only the Fab portion of the antibody is effective in targeting the antigen [72], and it may even hold some advantages over using the complete antibody. In one study, researchers investigated using liposomes conjugated with Fab fragments of Cetuximab to deliver Oxaliplatin to tumor tissue. It was found that conjugating Fab fragments increased drug accumulation in the tumor almost twofold over liposomes conjugated with the entire Cetuximab antibody [73]. The same trend is seen in other experiments [74], and there are some theories as to why this pattern is observed. Using the whole antibody may lead to higher immunogenicity, larger particle sizes, and lower efficacies than liposomes conjugated with Fabs [75,76,77]. Consequently, the current trend in antibody-conjugated liposomes is using the Fab portion of the antibody.

Overall, antibody-conjugated liposomes have a long way to go to show clearly that their added therapeutic value over conventional liposomes is worth the added cost of development. The use of Fabs is gaining momentum, and the data can be encouraging. However, the inconsistent performance in clinical trials raises some concerns in the short term.

### 3.2. Peptides

Peptides as bioactive moieties have been around for decades, and these short amino acid chains are known to be effective in the treatment of diseases. Their structure and size allow them to modulate protein-protein interactions, a crucial interface for many diseases [78]. The commercialization of insulin in 1923 marked the beginning of peptides for therapeutic use [79], and the introduction of recombinant DNA technology in the 1970s accelerated their development into approved products. They are a popular choice of molecule for disease mechanism-based drug discovery, and there have been over 30 different peptides approved by the FDA since 2000 [80]. This is because, compared to traditional small-molecule drugs, peptides often exhibit higher specificity, possess higher bioactivity, and cost less money to develop [81]. They are also highly versatile, with their applications including areas such as hormone supplementation [82,83], antimicrobial treatment [84,85,86], and vaccine development [87,88,89].

With their wide range of therapeutic potential and high specificity, it is no surprise that peptides have been conjugated to the surface of liposomes for targeted drug delivery (Figure 4). It is often the case that certain receptors are overexpressed within diseased tissue. Some examples include the overexpression of human epidermal growth factor receptor 2 (HER2) in aggressive forms of breast cancer [90], p21-activated kinase 1 (PAK-1) in prostate cancer [91], and CD13 in tumor vasculature [92]. Peptides can be formulated to specifically bind to these receptors. If these peptides are conjugated to the surface of liposomes, then the encapsulated drug can be delivered to the diseased tissue efficiently. There is much research dedicated to this therapeutic area. In one example, a cyclic arginine–glycine–aspartic acid-tyrosine–lysine peptide (cRGDyk) was used to target αvβ3 integrin, a receptor highly expressed in the case of bone metastasis. Liposomes conjugated with cRGDyk were internalized by tumor cells at a rate over 3-fold higher than untargeted liposomes, and they were over 88% more cytotoxic than the free drug. In vivo, the targeted liposomes were shown to improve circulation time, bone lesions, and overall survival over the untargeted liposomes [93]. Additionally, cRGD has been shown to be an effective targeting mechanism for delivering microRNA to cancer stem cells and breast carcinoma cells. cRGD-conjugated liposomes demonstrated an increased accumulation of the microRNA and the suppressed proliferation of the cancer cells as compared to untargeted liposomes [94]. Peptide-conjugated liposomes can also be effective for treating diseases other than cancer. Liposomes carrying azithromycin have been conjugated with an antibacterial peptide DP7-C to target surface receptors on methicillin-resistant Staphylococcus aureus (MRSA). Compared to untargeted liposomes, these targeted liposomes showed enhanced efficacy, increased immune activation, and showed no signs of mammalian cytotoxicity [95]. There is a plethora of other receptors that are used as targets for peptide-conjugated liposomes [96], and there are even more that have yet to be defined. For this reason, there are research efforts across many different fields focusing on elucidating the role that different receptors play in disease mechanisms. If the importance of a specific receptor can be established, a peptide-targeted therapy can conceivably be developed.

The laboratory-scale development of these targeted liposomes has shown promise. However, there are still some considerations that need to be more thoroughly researched for peptide-conjugated liposomes to become commonly approved drug products. Firstly, the accurate quantification of peptides on the liposome surface is difficult and not yet optimized [97]. This may lead to harsh criticism during any regulatory submission due to concerns over significant batch-to-batch variation. Additionally, if peptides are conjugated to the liposome surface in very high densities, the liposomes are prone to aggregation, leading to an increased particle size and accelerated clearance from the blood [36]. Even if the large-scale formulation and characterization can be perfected, the storage of the liposomes is still a challenge. Refrigeration is commonly used, but drug leakage can occur over time [98]. Overall, there is reason to be optimistic about these targeted nanoparticles, but there are still problems that need to be solved.

### 3.3. Folate

Folate, also known as vitamin B9, plays an interesting and important role in the development and progression of cancer. Folate is an essential molecule for the methylation of DNA and RNA [99]. Consequently, it is crucial that someone receives ample amounts of folate in their diet to enable proper DNA and RNA synthesis and prevent instances of cancer. Indeed, it has been suggested that increased folate intake is associated with lower rates of cancer [100,101]. However, folate can actually be detrimental if cancer is already present. Due to the high proliferation of malignant tumors, there is an increased requirement for DNA synthesis, and tumor cells of many different cancers will overexpress the receptor for folate [102]. As a result, increased folate within the tissue will bind to these receptors and encourage tumorigenesis [103].

Because of their importance in the proliferation of tumor cells, folate receptors are a common target for cancer therapies. Folate can be conjugated to the surface of liposomes as a means of targeting the overexpressed folate receptors on tumors (Figure 4). There are many examples of folate being used for targeted liposomes in the published literature and researchers have observed a significantly increased cellular uptake, cytotoxicity, and specificity of the targeted liposomes in vitro [104,105,106].

Despite these encouraging results, there are some concerns over the in vivo performance of folate-targeted liposomes. Firstly, the observed pharmacokinetics are often underwhelming. It seems that the intravenous administration of folate-conjugated liposomes may reduce circulation time and bring no improvement to the accumulation of the drug in the tumor as opposed to a conventional liposome [105,107,108]. This of course defeats the purpose of developing a targeted therapy. A second issue with the in vivo results of folate liposomes is the inefficiency of drug unloading once the liposome has reached its target [109]. This limitation reduces the overall potency. These two concerns damage the outlook of folate liposomes for clinical use, but there are some strategies that may help. For improved pharmacokinetics and more efficient drug unloading, a researcher may consider local administration [105] or pH-sensitive liposomes [110], respectively, as solutions. These strategies have shown promise, but further data are needed to support the preferential use of folate-targeted liposomes.

### 3.4. Aptamer

Aptamers are single stranded sequences of structured RNA or DNA that can interact with binding sites on a wide range of molecules [111]. While nucleotides are typically thought of as a means of storing genetic information, aptamers are proof of an untraditional function of nucleotides that very closely mimics antibodies and other ligands. The defined structure of an aptamer allows the molecule to bind specifically to corresponding active sites, giving them the nickname ‘chemical antibody’. The research interest in aptamers is fairly new, with the first FDA-approved aptamer drug product, pegaptanib (Macugen^®^), reaching the market in 2004 [112]. Since 2004, though, there has been only one additional aptamer approval, and the overall speed of development for aptamer-based drug products seems to have stalled slightly compared to other molecules like peptides and antibodies [113]. However, aptamers have shown promise due to their versatility, low cost, scalable production, and little to no immunogenicity [114].

**Figure 5:**
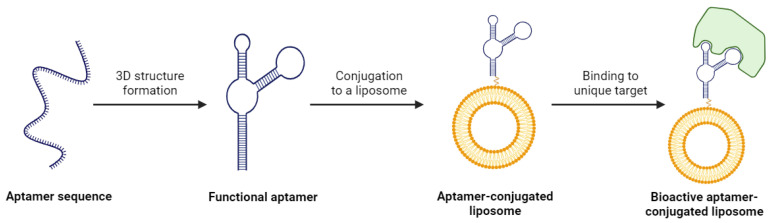
The process of creating a bioactive aptamer-conjugated liposome. Created with BioRender.com on 24 March 2024.

One of the major appeals of using aptamers for liposome functionalization is their wide range of therapeutic targets. Aptamers are identified using a technique known as the systematic evolution of ligands by exponential enrichment (SELEX) [115]. This process exposes a desired target to a large library of diverse aptamers and isolates the aptamers that display the highest affinity towards the target. The aptamers demonstrating affinity are multiplied through PCR, and the experiment is repeated several times. The result of SELEX is an aptamer with exceptional selectivity for the target, which could be a protein, small molecule, or even a whole cell (Figure 5) [116]. The versatility of aptamer binding and this preferential selection of candidates means it is possible to develop a ligand for a target whose surface is either not entirely characterized or is very complex in nature. This is a massive advantage over antibodies or other ligands, and it creates the opportunity for a unique mechanism of targeted drug delivery.

Aptamer-functionalized liposomes are far from FDA approval. However, the unconvincing in vivo results of antibody-conjugated liposomes leave researchers looking for alternatives, and aptamers offer several advantages over antibodies that have led to an increased interest. Currently, aptamer-conjugated liposomes have been used in research to selectively bind and deliver drugs to a variety of tissues [117], including cancer cells [118,119,120,121], corneal cells [122], muscle tissue [123], and intestinal epithelia [124]. For the most part, these liposomes have fared well during in vivo studies as compared to antibody-conjugated liposomes. A search on PubMed or Google Scholar will reveal a significant number of studies involving aptamer-functionalized liposomes over the last few years, indicating their growing popularity. That being said, there is an industry familiarity with antibodies and peptides, and trends in medicine can be slow to take hold. Despite many of the drawbacks of antibodies [125], it may be many years until an aptamer-conjugated liposome is FDA approved.

## 4. pH-Sensitive Liposomes

The microenvironment of a tumor is often characterized as quite hostile and significantly different than the physiological conditions of healthy tissue. Both the interstitial fluid surrounding cancerous tumors and the intracellular environment of tumor cells exhibit a pH significantly lower than the normal pH of 7.4–7.5 in healthy tissue and blood [126]. Liposomes can be formulated to release their encapsulated drug at these sites of lower pH when pH-responsive components are included in the lipid bilayer. This targeting strategy results in site-specific delivery and significant drug accumulation, which is a major advantage as compared to the conventional delivery of chemotherapeutic drugs [127].

The main components of pH-sensitive liposomes include 1,2-dioleoyl-sn-glycero-3-phosphoethanolamine (DOPE), a non-bilayer lipid that acts as the destabilizing agent in low pH due to polymorphic changes, and weakly acidic amphiphiles such as cholesteryl hemisuccinate (CHEMS), phosphatidylglycerol (PG), phosphatidylserine (PS), oleic acid, and linoleic acid [126,127,128,129,130]. When these liposomes come into contact with the acidic tumor microenvironment, the amphiphilic lipids’ carboxylic groups are protonated, leading to reduced stability and the formation of complex non-spherical structures [127]. This conformational change causes DOPE to transition from its stable lamellar phase into its inverted hexagonal phase, thus destabilizing the liposome bilayer complex [128]. As a result, the liposome undergoes endocytic uptake by the bound tumor cells [127,129].

Aside from reduced offsite toxicity, the primary advantage of pH-sensitive liposomes is the intracellular release of the therapeutic agent. While not fully understood, there are a few hypotheses for how the liposome releases the agent intracellularly rather than allowing it to be degraded by lysosomes through the endocytic pathway [127,129]. The first theory is that the liposome destabilizes the surrounding endosome by generating pores in its membrane, allowing the drug to diffuse through. A second theory claims that the liposome destabilizes fully within the endosome, and the free drug diffuses naturally through the intact endosomal membrane. A third theory suggests that the liposome fuses with the endosome’s membrane and releases the drug intracellularly [127]. Ultimately, as long as the drug is indeed released intracellularly, pH-sensitive liposomes will be considered clinically relevant. However, the true method of release should be elucidated for a full understanding of the mechanism of action.

The applications of pH-sensitive liposomes include the delivery of chemotherapies, genetic therapy agents, and immunogenic compounds for vaccines. In the treatment of cancerous tumors, the reaction of pH-sensitive liposomes to the acidic microenvironment allows for high specificity and reduced adverse effects. This form of liposomal drug delivery has high potential in the treatment of cancer as well as in the combinational formulation of multi-layered liposome–polymer nanoparticles for further targeting [131].

## 5. Temperature Sensitive Liposomes

Another strategy for targeted liposomal therapy is the use of temperature-sensitive liposomes with phase transition temperatures (Tm) slightly above physiological temperatures. The Tm of a lipid is the temperature above which the lipid shifts from a gel-like solid state to a disorderly liquid crystalline phase [132]. With lipids in this liquid phase, a liposome becomes much less rigid and therefore more permeable. These liposomes will remain stable at body temperature for long enough to reach the diseased tissue, whether it be by active targeting or the EPR effect. Then, an external stimulus is used to heat the diseased tissue. This temperature increase will heat the liposome above its Tm and trigger the release of the encapsulated drug molecules through the fluidic membrane. In this way, the drug release rate can be markedly improved over traditional liposomes.

Yatvin et al. were the first to have reported the formulation of temperature-sensitive liposomes in 1978 [133], using dipalmitoyl phosphatidylcholine (DPPC) as the primary lipid. DPPC was a suitable choice of lipid for temperature-sensitive liposomes due to its Tm of 41 degrees Celsius, but the formulation has been improved upon since. Using multiple types of lipids in a single liposome may be better than DPPC alone, as the increased heterogeneity leads to imperfect alignment in the membrane, resulting in an increased drug release [134]. Other commonly used lipids include distearoyl phosphocholine (DSPC) and hydrogenated soy phosphocholine (HSPC). Furthermore, there is evidence showing that the inclusion of other molecules in the membrane such as lysolipids [135,136,137] or polymers [138,139,140] may be beneficial for lowering Tm and improving drug release as well.

While the majority of research for temperature-sensitive liposomes has been towards the formulation of the liposomes themselves, the method by which clinicians provide localized heat is also of great importance. There are several different methods of administration, and the type of tumor may dictate which options are feasible. For example, invasive heating techniques such as percutaneous radiofrequency or microwave ablation are already in clinical use for cancer and have an impressive penetration depth. However, they can be expensive, require imaging-assisted surgery, and carry a risk for tumors near vital organs [141,142,143,144]. On the other hand, providing near-infrared (NIR) light to the diseased tissue is a noninvasive technique that increases the local temperature without damaging skin, but it has poor tissue penetration (3 cm), limiting its applications [145,146,147]. Perhaps the most promising technology for local heating is the use of high-intensity focused ultrasound (HIFU). This is a noninvasive technique that uses pinpointed high-frequency sound waves to heat a portion of tissue with high specificity. HIFU is a safe, cost-effective technique that has shown promise in its use with temperature-sensitive liposomes [148,149,150]. Ultimately, further clinical studies are needed to fully characterize each of these technologies’ efficacy and risk profile when used with thermosensitive liposomes.

Temperature-sensitive liposomes are presented as a solution to a problem often encountered in traditional liposomes. Delivering a liposome to the desired site can be accomplished through any of the methods discussed in this review. However, the drug still needs to be released by the liposome and received by the target for the treatment to be effective. This is often the source of disappointing in vivo results, leading to interest in investigating the release mechanism of liposomal drug products [151,152]. Using temperature-sensitive liposomes provides a known mechanism of release, giving researchers a better understanding of the drug product they are developing. While there are no temperature-sensitive liposomes currently approved, many have reached clinical stages of development [142], and they remain an intriguing prospect in targeted therapy.

## 6. Future Perspectives

As mentioned, the progress of novel targeting techniques is often hindered by less-than-impressive in vivo results. Most likely, these disappointing results are due to a lack of a complete understanding surrounding the relevant biological system. An eagerness to develop a marketable product can sometimes get in the way of fully characterizing the mechanisms behind the targeting and release of liposomal drugs. Doxil^®^, for example, was commercially available for years before researchers began to understand why the ABC phenomenon was not observed after its repeated administration [56,57]. In many cases, there are mechanistic questions about the liposomes’ interaction with the components of our blood and immune system that have yet to be answered. For further development of the novel delivery strategies covered in this review, further research is needed to characterize both the targeting pathways themselves, as well as the physiological conditions of the targeted tissue.

Moreover, there are also concerns regarding a liposome’s long-term stability. Liposomes have a high free energy on their surface due to their high surface area to volume ratio [153]. This can lead to physical instabilities such as aggregation or particle fusion, both of which can induce an increase in particle size and drug leakage [154]. Furthermore, chemical instabilities such as the degradation of surface-conjugated ligands are also detrimental to the targeting mechanism. There is great interest in developing a method of storage that will reduce these harmful reactions. Notably, lyophilization has been effective in increasing the shelf-life of nanoparticulate drug products [155]. The lyophilization process creates a solid powder from a liquid solution, and the powdered liposomes better retain their size characteristics and encapsulated drug throughout storage [156]. Ambisome^®^ and Visudyne^®^ are two examples of lyophilized liposomal drug products that have already been FDA-approved [156]. Despite the regulatory success, however, there are still many questions surrounding lyophilization. The optimal process parameters seem to vary with each lipid formulation, presenting a massive challenge to formulators aiming to use the process. Additionally, the hydrophilicity of the encapsulated drug and the size of the nanoparticle itself both can lead to instabilities during lyophilization [157,158]. Extensive preclinical experimentation is needed to definitively determine if lyophilization will work for a given liposome formulation, making it a potentially timely and costly endeavor. However, in some cases, these efforts may be necessary for extending the long-term stability and bringing targeted liposomal drug products to market.

In addition to storage instabilities, targeted liposome manufacturers also face the challenge of very limited guidance from the FDA. The rate of the lab-scale development of targeted liposomes has drastically outpaced the FDA’s reaction time to the new technology [159]. While there is some guidance provided for liposomal products in general [160], these documents do not adequately cover the novel targeting strategies discussed in this review. Therefore, the drug sponsor bears the responsibility of providing sufficient data that prove the novel carrier mechanism is both safe and effective. In other words, many targeted liposomes face significant regulatory hurdles before approval.

Yet, great progress has been made in the clinic for many of these products. As more data are published showing the efficacy of these targeted liposomes, more attention will be given to the logistical issues preventing their further development, such as storage instabilities and regulatory challenges. When these problems are solved, novel targeted liposomes will become much more therapeutically relevant. Many are already patented or progressing through clinical trials, as seen in Table 2 and Table 3.

## 7. Conclusions

Liposomes are well-established as one of the most effective nanoparticles for drug delivery. Their versatility has provided researchers with a plethora of options to achieve the specific and efficient delivery of therapeutics. The innate characteristics of liposomes provide a natural targeting system through the EPR effect, while active targeting techniques and external stimuli can be used to further enhance specific targeting and release (Table 4). For many of the targeting strategies, the in vivo data are still not quite up to the standard needed to create excitement around the delivery method. However, the mechanisms for these targeted therapies are fundamentally sound and show promise with additional optimization. Indeed, their proven biocompatibility and efficacy demonstrated in much of the written literature is reason enough to be optimistic about the development of targeted liposomal formulations. With continued investment, these products can be further characterized to overcome the current challenges to their approval.

## Figures and Tables

**Table 1:** A summary of FDA-approved liposomal drug products as of 2023.

Marketed Name	API	Approval Year	Indication	Reference
Doxil^®^	Doxorubicin	1995	Ovarian cancer, Kaposi’s sarcoma, multiple myeloma	[7]
DaunoXome^®^	Daunorubicin	1996	Kaposi’s sarcoma	[7]
Onivyde^®^	Irinotecan hydrochloride trihydrate	1996	Pancreatic adenocarcinoma	[7]
AmBisome^®^	Amphotericin B	1997	Breast cancer	[7]
DepoCyt^®^	Cytarabine	1999	Lymphomatous meningitis	[7]
Visudyne^®^	Verteporphin	2000	Age-related macular degeneration	[7]
DepoDur^®^	Morphine sulfate	2004	Pain management	[7]
Exparel^®^	Bupivacaine	2011	Anesthesia	[7]
Marqibo^®^	Vincristine	2012	Leukemia	[7]
Shingrix^®^	Recombinant varicella zoster virus glycoprotein E	2017	Shingles	[7]
Vyxeos^®^	Daunorubicin + cytarabine	2017	Leukemia	[7]
Arikayce^®^	Amikacin	2018	Lung infection	[7]
Onpattro^®^	Patisiran	2018	Hereditary transthyretin-mediated amyloidosis	[7]
Comirnaty^®^	mRNA	2021	COVID-19	[7]
Spikevax^®^	mRNA	2022	COVID-19	[7]

**Table 2:** Some examples of targeted liposomes progressing through preclinical and clinical trials currently.

Clinical Phase	Targeting Mechanism	Intervention	Indication	Name or Clinical Trial ID	Reference
Preclinical	Aptamer and PEG	Doxorubicin	Cancer	Syl3c-PLD	[161]
	Aptamer and PEG	Doxorubicin	Cancer	ER-lip	[118]
	pH Sensitive	Docetaxel, SIRT1 shRNA	Breast cancer	DTX-lipoplex	[162]
	pH Sensitive and PEG	Gemcitabine Curcumin	Pancreatic cancer	PSL	[163]
	pH Sensitive, Folate, and PEG	Doxorubicin	Breast Cancer	SpHL-DOX-Fol	[164]
	Folate and PEG	BIM-S plasmid	Non-small lung cancer	F-PLP/pBIM	[165]
	Peptide (cRGD) and PEG	Doxorubicin	Cancer	cRGD-KK-SG/Dox-PEGylated liposomes	[166]
	Antibody (anti-CD19 and anti-CD20 monoclonal antibodies) and PEG	Doxorubicin	Breast cancer	DOPC-PLD	[167]
	Peptide (LinTT1) and PEG	Doxorubicin and sorafenib	Breast cancer	LinTT1 Liposome	[168]
Phase 1	Thermosensitive and PEG	Doxorubicin with HIFU	Breast cancer	NCT03749850	[169]
	Thermosensitive and PEG	Doxorubicin with HIFU	Liver tumor	NCT02181075	[170]
	Thermosensitive and PEG	Doxorubicin with HIFU	Pediatric solid tumors	NCT02536183	[170]
Phase 2	Thermosensitive and PEG	Doxorubicin with microwave hypothermia	Breast cancer	NCT00826085	[170]
	Thermosensitiveand PEG	Doxorubicin with radiofrequency ablation	Colon cancer and liver metastasis	NCT01464593	[170]
	Antibody (Cetuximab Fab fragment)	Doxorubicin	Breast cancer	NCT02833766	[170]
Phase 3	Thermosensitive and PEG	Doxorubicin with radiofrequency ablation	Hepatocellular carcinoma	NCT02112656	[170]

**Table 3:** Some examples of US patents that describe targeted liposomes.

Patent Number	Title	Description	Reference
US201916592263A	Nanoparticle arsenic–platinum compositions	This patent covers the co-encapsulation of active forms of arsenic and platinum drugs into liposomes and methods of using these liposomes for the diagnosis and treatment of cancer. Specifically, these liposomes are stable at physiological temperatures and release the encapsulated drug at low pH.	[171]
US2017022629W	Treating ephrin receptor a2 (Epha2) positive cancer with targeted docetaxel-generating nano liposome compositions	This patent describes docetaxel-carrying liposomes conjugated with both PEG and an antibody for EphA2. They are documented to release docetaxel at physiological pH	[172]
US6339069B1	Peptide–lipid conjugates, liposomes and liposomal drug delivery	This patent describes a liposome formulated with peptide-conjugated lipids that upon contact with the corresponding peptidase will destabilize and release the encapsulated drug	[173]
US20020025313A1	Targeting of liposomes to the blood–brain barrier	This patent covers targeted liposomes for the delivery of drugs to the brain using conjugated antibody fragments. The listed targets include transferrin receptor, insulin receptor, insulin-like growth factor-1, insulin-like growth factor-2, and more.	[174]
US9833464B2	Target-aiming drug delivery system for diagnosis and treatment of cancer containing liposome labeled with peptides which specifically targets interleukin-4 receptors, and manufacturing method thereof	This patent covers the liposomal drug delivery for diagnosis and treatment of cancer using conjugated peptides that target interleukin-4 receptors	[175]
US10023656B2	Methods of treating inflammation using IL-17A and IL-17F cross-reactive monoclonal antibodies	This patent describes the use of antibodies for blocking, reducing, antagonizing, or neutralizing the activity of interleukin-17A and iterleukin-17F. The patent includes methods of using these antibodies, including liposomal vehicles.	[176]

**Table 4:** The advantages and limitations of the discussed targeting mechanisms are summarized.

Targeting Strategy	Mechanism	Advantages	Limitations	References
Passive Targeting	EPR Effect	Natural phenomenon Can be further enhanced or combined with other therapies (e.g., phototherapy)	Limited specificity Magnitude of effect varies by characteristics of tumor	[33,177]
	PEGylation	Prolonged circulation Less drug leakage and instabilities	May induce ABC phenomenon	[38,40,47]
Active Targeting	Antibodies	High specificity Fab portions can be used for possibly better pharmacokinetics	Possible immunogenicity Mixed in vivo results	[63,67,72,75]
	Peptides	Versatile applications Low immunogenicity	Stability and storage concerns	[36,82,84,86]
	Folate	High affinity for folate receptors in many cancers	Not applicable to all tumors or diseases Pharmacokinetic concerns	[102,104,105]
	Aptamers	Wide range of targets Low immunogenicity	Stability concerns Unfamiliar to the industry	[111,113,114]
External Cues	pH Sensitive	Low off-target toxicity Intracellular delivery	Premature drug release	[127,178]
	Temperature-Sensitive	Effective drug unloading Manual release	Limited applicability	[134,142]
